# Supplementary material for: Nuclear genetic modulation of tissue-specific mitochondrial RNA processing contributes to common disease risk
Source: Nat Commun. 2026 Apr 30;17:5899. doi: 10.1038/s41467-026-72649-5 (PMC13338043; doi:10.1038/s41467-026-72649-5)
Supplement: Supplementary file 1 — Description of Additional Supplementary Files [file 41467_2026_72649_MOESM1_ESM.pdf]

Description of Additional Supplementary Files

Supplementary Data 1: Sample sizes for each tissue and inflation factors for eQTL mapping of each gene in each tissue.

Supplementary Data 2: Nuclear genetic variants associated with mtDNA-encoded transcript abundance.

Supplementary Data 3: For each peak genetic variant associated with a gene encoded in mtDNA, the table shows the asscoiation between that peak genetic variant and all genes in mtDNA in the same tissue.

Supplementary Data 4: For each peak genetic variant associated with a gene encoded in mtDNA, the table shows the asscoiation between that peak genetic variant and the same mtDNA gene across all tissues.

Supplementary Data 5: Nuclear genetic variants associated with mtDNA-encoded transcript abundance at FDR 5%.

Supplementary Data 6: mtDNA genetic variants associated with mtDNA-encoded transcript abundance.

Supplementary Data 7: Binary disease traits in UK Biobank, with codes, descriptions and case numbers

Supplementary Data 8: Diseases in UKBB that are significantly associated with predicted mtDNA-encoded transcript abundance after Bonferroni correction.

Supplementary Data 9: Quantitative traits in UK Biobank, together with definitions and details on how data were normalised and tested against mtDNA transcript abundance.

Supplementary Data 10: Quantitative traits in UKBB that are significantly associated with predicted mtDNA-encoded transcript abundance after Bonferroni correction.

Supplementary Data 11: TWAS results comparing models for each mtDNA-encoded gene in each tissue with five common age releated diseases.

Supplementary Data 12: Direct associations between top SNPs in each mtDNA transcript abundance prediction model that is significantly associated with a binary disease trait.

Supplementary Data 13: Direct associations between top-weighted SNPs from mtDNA transcript abundance prediction models that are significantly associated with quantitative traits.
