# Supplementary material for: Nuclear genetic modulation of tissue-specific mitochondrial RNA processing contributes to common disease risk
Source: Nat Commun. 2026 Apr 30;17:5899. doi: 10.1038/s41467-026-72649-5 (PMC13338043; doi:10.1038/s41467-026-72649-5)
Supplement: Supplementary file 3 — Reporting Summary [file 41467_2026_72649_MOESM3_ESM.pdf]

nature portfolio

Corresponding author(s): Alan Hodgkinson
Last updated by author(s): Apr 7, 2026

Reporting Summary

Nature Portfolio wishes to improve the reproducibility of the work that we publish. This form provides structure for consistency and transparency in reporting. For further information on Nature Portfolio policies, see our Editorial Policies and the Editorial Policy Checklist.

Statistics

For all statistical analyses, confirm that the following items are present in the figure legend, table legend, main text, or Methods section.

n/a	Confirmed	
[ ]	[x]	The exact sample size (*n*) for each experimental group/condition, given as a discrete number and unit of measurement
[ ]	[x]	A statement on whether measurements were taken from distinct samples or whether the same sample was measured repeatedly
[ ]	[x]	The statistical test(s) used AND whether they are one- or two-sided<br>*Only common tests should be described solely by name; describe more complex techniques in the Methods section.*
[ ]	[x]	A description of all covariates tested
[ ]	[x]	A description of any assumptions or corrections, such as tests of normality and adjustment for multiple comparisons
[ ]	[x]	A full description of the statistical parameters including central tendency (e.g. means) or other basic estimates (e.g. regression coefficient) AND variation (e.g. standard deviation) or associated estimates of uncertainty (e.g. confidence intervals)
[ ]	[x]	For null hypothesis testing, the test statistic (e.g. *F*, *t*, *r*) with confidence intervals, effect sizes, degrees of freedom and *P* value noted<br>*Give P values as exact values whenever suitable.*
[x]	[ ]	For Bayesian analysis, information on the choice of priors and Markov chain Monte Carlo settings
[x]	[ ]	For hierarchical and complex designs, identification of the appropriate level for tests and full reporting of outcomes
[ ]	[x]	Estimates of effect sizes (e.g. Cohen's *d*, Pearson's *r*), indicating how they were calculated

Our web collection on statistics for biologists contains articles on many of the points above.

Software and code

Policy information about availability of computer code

Data collection	No new raw data was generated in this project. All data was downloaded from protected repositories.
Data analysis	Data was analysed via a collection of standard genomics software and custom code. Software used: SAMtools (v1.14), STAR (v2.7.6), RNAseQC (v2.4.2), Trim Galore (v0.4.0), BWA-MEM (v0.7.17), Mutserve (v2.0.0-rc13), PEER, PLINK (v1.9), Eigensoft (v8.0.0), FUSION. Custom code used for analyses is available here: https://github.com/AJHodgkinson/mitoX

For manuscripts utilizing custom algorithms or software that are central to the research but not yet described in published literature, software must be made available to editors and reviewers. We strongly encourage code deposition in a community repository (e.g. GitHub). See the Nature Portfolio guidelines for submitting code & software for further information.

Data

Policy information about availability of data

All manuscripts must include a data availability statement. This statement should provide the following information, where applicable:
- Accession codes, unique identifiers, or web links for publicly available datasets
- A description of any restrictions on data availability
- For clinical datasets or third party data, please ensure that the statement adheres to our policy

GTEx protected data are available through the database of Genotypes and Phenotypes (dbGaP) (accession no. phs000424.v8) and public-access data are available on the GTEx Portal (www.gtexportal.org). UK Biobank data is available through https://www.ukbiobank.ac.uk, and was accessed under project number 46360.

Genetic score files and summary statistics for mtDNA-encoded transcript abundance across 49 tissue types are available via Github (https://github.com/AJHodgkinson/mitoX).

Research involving human participants, their data, or biological material

Policy information about studies with human participants or human data. See also policy information about sex, gender (identity/presentation), and sexual orientation and race, ethnicity and racism.

Reporting on sex and gender	Sex was used as a covariate in most analysis, and was determined by phenotype #31 in UK Biobank data.
Reporting on race, ethnicity, or other socially relevant groupings	Final analyses were conducted on 'European' ancestry samples. These were determined in GTEx by clustering samples on the first two genetic principle component axes, and in UK Biobank by using data returned (2442) by the Pan-UKBB analysis.
Population characteristics	Age, sex and genetic principle components were used as covariates in association analyses.
Recruitment	NA
Ethics oversight	The project was approved by the UK Biobank (application number 46360) and GTEx data access committees, and all analyses were conducted in accordance with the ethical approvals under which the data were originally collected. The Research Ethics Office of King's College London gave ethical approval for this work (LRS-18/19-10868).

Note that full information on the approval of the study protocol must also be provided in the manuscript.

Field-specific reporting

Please select the one below that is the best fit for your research. If you are not sure, read the appropriate sections before making your selection.

- [x] Life sciences
- [ ] Behavioural & social sciences
- [ ] Ecological, evolutionary & environmental sciences

For a reference copy of the document with all sections, see nature.com/documents/nr-reporting-summary-flat.pdf

Life sciences study design

All studies must disclose on these points even when the disclosure is negative.

Sample size	Sample sizes were determined by available expression, genetic and phenotype data in GTEx and UKBB.
Data exclusions	GTEx raw fastq files were aligned to a reference genome. After alignment, samples that had an intergenic mapping rate >30%, an overall base mismatch rate >1%, a ribosomal RNA mapping rate >30%, total reads < 5,000,000 or had zero reads for any ribosomal or protein coding gene encoded within mitochondrial DNA (mtDNA) were removed using in house scripts and RNAseQC (v2.4.2). UK Biobank data were filtered as per pre-defined outlier metrics. Full details are available in Supplementary table 9.
Replication	mtDNA transcript abundance prediction models were tested by 1) leaving 20% of GTEx data out as a testing set, 2) Independent vaildation in three external datasets.
Randomization	Individuals in UK Biobank were split into case/control for disease phenotypes, or had quantitative measurements available for quantitative analyses.
Blinding	Blinding is not relevant here - association testing was performed for disease and quantitative traits.

Reporting for specific materials, systems and methods

We require information from authors about some types of materials, experimental systems and methods used in many studies. Here, indicate whether each material, system or method listed is relevant to your study. If you are not sure if a list item applies to your research, read the appropriate section before selecting a response.

Materials & experimental systems

n/a	Involved in the study
x	Antibodies
x	Eukaryotic cell lines
x	Palaeontology and archaeology
x	Animals and other organisms
x	Clinical data
x	Dual use research of concern
x	Plants

Methods

n/a	Involved in the study
x	ChIP-seq
x	Flow cytometry
x	MRI-based neuroimaging

Plants

Seed stocks	*Report on the source of all seed stocks or other plant material used. If applicable, state the seed stock centre and catalogue number. If plant specimens were collected from the field, describe the collection location, date and sampling procedures.*
Novel plant genotypes	*Describe the methods by which all novel plant genotypes were produced. This includes those generated by transgenic approaches, gene editing, chemical/radiation-based mutagenesis and hybridization. For transgenic lines, describe the transformation method, the number of independent lines analyzed and the generation upon which experiments were performed. For gene-edited lines, describe the editor used, the endogenous sequence targeted for editing, the targeting guide RNA sequence (if applicable) and how the editor was applied.*
Authentication	*Describe any authentication procedures for each seed stock used or novel genotype generated. Describe any experiments used to assess the effect of a mutation and, where applicable, how potential secondary effects (e.g. second site T-DNA insertions, mosiacism, off-target gene editing) were examined.*
