# Supplementary material for: Nuclear genetic modulation of tissue-specific mitochondrial RNA processing contributes to common disease risk
Source: Nat Commun. 2026 Apr 30;17:5899. doi: 10.1038/s41467-026-72649-5 (PMC13338043; doi:10.1038/s41467-026-72649-5)
Supplement: Supplementary file 4 — Transparent Peer Review file [file 41467_2026_72649_MOESM4_ESM.pdf]

nature portfolio

# Peer Review File

# Nuclear genetic modulation of tissue-specific mitochondrial RNA processing contributes to common disease risk

Corresponding Author: Dr Alan Hodgkinson

**This file contains all reviewer reports in order by version, followed by all author rebuttals in order by version.**

Version 0:

Reviewer comments:

Reviewer #1

(Remarks to the Author)
General

The study analyzed over 15,000 RNA-seq samples from GTEx alongside matched whole-genome sequencing to investigate how nuclear and mitochondrial genetic variants influence mtDNA-encoded transcript abundance. They identified 25 significant nuclear genetic variants (trans-eQTLs) associated with mitochondrial gene expression, most mapping to genes involved in mitochondrial RNA processing. They also found 15 cis-eQTLs within mtDNA itself. Using these findings, they built genetic prediction models of mtDNA transcript levels, and used these predictions to test for associtions with quantitative traits and diseases in UK Biobank participants of European ancestry, indicating tissue specificity in mtDNA gene expression effects on complex traits and diseases. This is a comprehensive and well-executed study that nicely links molecular-level associations with complex phenotypes. I have the following comments:

1. mtDNA cis-eQTLs: most of the associations found in the mtDNA cis-eQTLs analyses have been found previously in Cai & Gomez-Duran et al 2020 Nature Medicine, please cite.

2. Prediction of mtDNA encoded gene expressions: It would be good to see a figure of prediction r2 of a gene vs its r2 across tissues i.e. whether the accuracy of gene expression prediction is related to how correlated its predicted expression is across tissues. This matters for downstream TWAS studies where predicted mtDNA gene expressions are assessed for association with phenotypes, when for traits like bilirubin, there are ~40 associations with predicted mtDNA genes, presumably the same mtDNA genes in many different tissues due to the high levels of correlations between the predicted values.

3. TWAS of mitochondrial encoded genes: How should we understand the TWAS signals - the authors tested whether these signals are likely pleiotropic effects of the nuclear SNPs on a cis-regualted nuclear gene or the mtDNA gene, but not whether the nuclear SNPs are associated with the complex traits themselves (through some other mechanism than the cis-regulated nuclear gene). Since most GWAS hits are not cis-eQTLs, it is possible that the nuclear SNPs regulate the complex traits in a way that is not through the mtDNA genes, but not its cis-eQTLs either. I suggest the authors, at the very least, list the association of the nuclear SNPs with the complex traits or disease in question and discuss the possibility there are other indirect mechanisms in play.

4. TWAS effect sizes: some of the TWAS effect sizes are extraordinarily small e.g. 0.0000000001309716 for association between ENSG00000198840 in Artery Aorta (blup model) for C reactive protein, which gave a P value of 4.89E-11 in 300K samples (numbers taken from Supp Table S7). This doesn't look right to me, the authors need to check this and convince me this is right. In any case, the authors need to put in standard errors for the effect sizes. Other TWAS signal have slightly more credible yet still really small effect sizes, e.g. ENSG00000198899 in AdrenalGland has a effect of -0.0026332210 on C_reactive_protein_CRP with a P value of 2.54E-17. One reason is of course, these are not standardised effect sizes, but raw effect sizes (i.e. gene expression and complex traits both not scaled to mean 0, sd 1). What this would mean is tiny effects on gene expression of mtDNA genes are significantly associated with complex traits. If this was true, the authors would need to write about this explicitly and provide a plausible biological explanation how this can be.

5. TWAS tissues of interest: For traits like bilirubin where there are many tissues with the same associations, some are unlikely to be mechanistically related to bilirubin like the brain tissues. In other words, Fig 4 represents the statistically significant findings, but is likely an inflated view of how much mtDNA genes affect complex traits. I would ask the authors to include another figure (4b?) that plots the number of mtDNA genes associated rather than the total number of associations, to account for the number of redundant associations due to between tissues mtDNA gene expression correlations.

6. Most of the mechanistic explanations given by the authors, for each group of traits associated with predicted mtDNA gene expression levels, goes along the lines of "this supports mtDNA involvement/influence on this trait/disease". This is fair enough given how little is known and the progress that is actively made by this paper to advance this. However, I would like to see a discussion section on actual mechanistic speculations how mtDNA genes may mediate complex traits and diseases, and an outlook for future investigations.

(Remarks on code availability)
Code is not complete on the github page, the scripts in the README do not match the name of the executable files, some executable files not present in github e.g. /scratch/grp/hodgkinsonlab/shared/GTEx_Data/Models/main.nf

Reviewer #2

(Remarks to the Author)
Summary:
The authors leverage tissue-specific RNA and genome sequencing datasets to identify genetic variants associated with mitochondrially-encoded gene expression and disease risk. They identify 25 variants associated with mtRNA expression, 21 of which link to 5 nuclear genes involved in mitochondrial processes. Notably, 8 of the 25 associations involve FASTKD4, a nuclear-encoded protein that regulates mitochondrial RNA stability. The authors build machine learning models to predict mtRNA expression across tissues, construct genetic score models for transcript abundance, and evaluate associations between predicted mtRNA expression and disease traits. They identify numerous trait-disease associations and explore quantitative trait associations using their genetic scores.

Overall, this work provides valuable new insights into the genetic regulation of mtRNA expression and its relationship to disease. However, several sections lack clarity, figures require further development, key results are missing from tables, and the overall take-home message needs to be articulated more clearly. More important methodological methods are not clearly explained. In parts, the lack of methods details has made it difficult to assess the quality of their analysis.

Major issues:
1. Incomplete presentation of variant effects across mitochondrial gene expression and in the affected nuclear genes.

For nuclear-encoded genes: The authors should demonstrate the effect of these variants on the expression levels of the associated nuclear genes. For example: Do the missense variants in FASTKD4 affect its total RNA levels? What about different FASTKD4 isoforms for the intronic variants? How do these changes explain the observed alterations in mitochondrial gene expression?

For mitochondrially-encoded genes: The manuscript does not clearly show which other mtDNA-encoded genes are affected by each identified variant. The authors state that "Considering all 350 possible variant–gene combinations (25 variants x 14 other mtDNA genes), we found that 58% showed nominal associations ($P<0.05$), suggesting a mixture of both broad and gene-specific regulatory effects." However, these results are not presented in either figures or supplementary tables. The authors have to show these results in the main figure and supplementary tables.

Moreover, the authors should relate their results to the literature on how effects in different nuclear encoded genes affect mtDNA expression. As an example, the authors could discuss how previous work support a model in which MTPAP and FASTKD4 can differentially affect different mtRNAs.
Regarding tissue-specific differences: It is not clear which biological conditions might give rise to the observed diversity in response to genetic variants. Are the differences across tissues related to differential effects on the expression levels of the associated nuclear genes? For example, do different tissues show different effects on mtRNA gene expression levels?

In Figure 1B the authors should provide a visual representation of statistical significance (e.g., p-values indicated by symbols or color intensity). Moreover, the authors do not explain why some tissues show increased MTATP6 levels (e.g., Colon) while others show reduced levels (e.g., Muscle) for the same variant. This directional variability across tissues requires explanation.
Finally, as different tissues have different levels of expression of individual mtRNAs (Mercer et al 2011), the authors should discuss how this tissue variability can affect their results. Moreover, they should consider this in their methods when comparing gene expression differences across tissues (see bellow).

2. Machine Learning Models: Insufficient methodological detail and integration with the biology discussed in the paper.

The machine learning section lacks critical details about model construction and interpretation. The authors state they used LASSO, Elastic Net, and BLUP to build predictive models, selecting genetic variants "based on different P-value thresholds." However, the Methods section does not clarify which features were used as model inputs, whether or how

features were scaled before training, how model optimization was performed and the final feature coefficients in each model. Moreover, since LASSO and Elastic Net are interpretable algorithms where feature importance relates directly to coefficients, it is essential to report which features are important and their relative weights.

In Figure 2C, the correlation between internal and external $R^2$ values appears to show two distinct populations of points. This pattern is not discussed.

The authors should expand on their interpretation of the results. The could explain how the results from the machine learning models relate to the downstream disease association analyses or the biology of the mtDNA expression.

3. Age-related disease associations: The section "Associations with age-related common diseases" discusses important findings but lacks accompanying figures or comprehensive tables. The authors should provide a figure or table summarizing these associations for easier interpretation.

4. RNA sequencing analysis
The RNA sequencing analysis lacks critical detail about how the libraries were constructed. In page 19 the authors say: “Second, although we model gene expression separately for each of the 15 mtDNA-encoded genes, mitochondrial transcription operates via polycistronic mechanisms. Some genes (e.g., MTATP6/8) are bicistronic, and MTND6, transcribed from the light strand, is not polyadenylated and thus should be underrepresented in GTEx poly-A+ data. Similarly, mitochondrial rRNAs are minimally adenylated and typically excluded from standard RNA-seq. Nevertheless, we find robust signals for several of these genes, which may reflect alternative processing (e.g., degradation fragments or longer precursor transcripts) and still carry informative biological signal.”

Are the GTEx libraries polyA-selected libraries? In that case, the authors should emphasize this point more strongly and clearly and tone down their conclusions. It is not clear whether the levels of these transcripts represents a difference in the library quality rather than a real biological difference.
How are the authors controlling for library depth differences across tissues? Do they observe any bias? Are they excluding outlier samples? PCA analysis and other quality metrics should be presented in a supplementary figure.

For the RNA sequencing analysis, the authors take alignment files, convert them back to fastq files, trim, and realign them. What is the reason for this? The authors do not have access to raw fastq files? This procedure can be problematic as part of the original data might be lost in the first alignment step, to which the authors’ approach is unaware of. Did the authors make sure their fastq files are similar to the raw data from GTEx? The authors should discuss this in their methods section.
Given that different tissues have different numbers of samples and RNA sequencing depth, the authors should perform a power analysis to understand how much of the difference they see across tissues might arise from this fact rather than a biological difference.

Minor issues:
The statement "These associations point to RNA processing, rather than transcription initiation alone, as a major determinant of mtRNA abundance" appears to overlap with findings from previous work. Please clarify the novel contribution here.

The authors highlight a number of genes known to function within the mitochondria, including FASTKD4, FASTKD5, PNPT1, and MTPAP which they claim are linked to associations listed in Table 1. The text lacks citation to any literature on these genes to support the author’s description of gene function. Citation would be especially useful for the statement “PNPT1 is responsible for the import of RNA into the mitochondria”, as import of RNA into human mitochondria is highly contested. Additionally, the text later refers to LONP1 as being involved in mitochondrial RNA processing, but does not comment on the known role of LONP1 as a mitochondrial protease.

(Remarks on code availability)

Reviewer #3

(Remarks to the Author)
I co-reviewed this manuscript with one of the reviewers who provided the listed reports. This is part of the Nature Communications initiative to facilitate training in peer review and to provide appropriate recognition for Early Career Researchers who co-review manuscripts.

(Remarks on code availability)
I reviewed the code used for RNA sequencing as this is my area of expertise. The script provided is a perl script that generates a bash script that runs the pipeline from bam to fast conversion to alignment and sorting. The scripts is well structured and clear for an individual that is familiar with these processes but it will benefit from more comments to help users less familiar with the general RNA sequencing analysis.

Version 1:

Reviewer comments:

Reviewer #1

(Remarks to the Author)
The authors have answered my concerns and I recommend acceptance. In reviewing the code again I see that summary statistics are not yet provided, and would urge the authors to make the summary statistics publicly accessible on paper acceptance and before publication.

(Remarks on code availability)
No summary stats, it says "to come"

Reviewer #2

(Remarks to the Author)
Publication will be recommended upon the following revisions.

Making SNP weights available in the data release is a welcome step, but Supplementary Table 13 lacks column descriptions. A brief legend should be added so future researchers can interpret these results.

The expanded Discussion addresses potential biological mechanisms in general terms but does not connect them to specific ML model results. The authors are encouraged to include one or two concrete examples linking a model outcome to a biological inference (e.g., which gene, in which tissue, motivates a given mechanistic interpretation).

Finally, if the primary contribution of this paper is a model demonstrating that mtRNA levels can be predicted from genetic information, then the full model with tissue-specific weights must be made publicly available in a usable form, ideally via GitHub with sufficient documentation to allow retraining and reuse. The current GitHub page is incomplete and does not provide a clear path for reproducing the results. This should be addressed before publication.

(Remarks on code availability)

Reviewer #3

(Remarks to the Author)
I co-reviewed this manuscript with one of the reviewers who provided the listed reports. This is part of the Nature Communications initiative to facilitate training in peer review and to provide appropriate recognition for Early Career Researchers who co-review manuscripts.

(Remarks on code availability)
The code for RNAseq analysis has now useful comments.

**Open Access** This Peer Review File is licensed under a Creative Commons Attribution 4.0 International License, which

permits use, sharing, adaptation, distribution and reproduction in any medium or format, as long as you give appropriate credit to the original author(s) and the source, provide a link to the Creative Commons license, and indicate if changes were made.
In cases where reviewers are anonymous, credit should be given to 'Anonymous Referee' and the source.
The images or other third party material in this Peer Review File are included in the article's Creative Commons license, unless indicated otherwise in a credit line to the material. If material is not included in the article's Creative Commons license and your intended use is not permitted by statutory regulation or exceeds the permitted use, you will need to obtain permission directly from the copyright holder.
To view a copy of this license, visit https://creativecommons.org/licenses/by/4.0/

We thank the reviewers for the thoughtful and constructive comments on our manuscript. Below, we provide a detailed, point-by-point response outlining all changes made. Importantly, while we have refined several aspects of the analysis and interpretation, the major findings and overall conclusions of the study remain unchanged.

REVIEWER COMMENTS

***Reviewer #1 (Remarks to the Author):***

General: The study analyzed over 15,000 RNA-seq samples from GTEx alongside matched whole-genome sequencing to investigate how nuclear and mitochondrial genetic variants influence mtDNA-encoded transcript abundance. They identified 25 significant nuclear genetic variants (trans-eQTLs) associated with mitochondrial gene expression, most mapping to genes involved in mitochondrial RNA processing. They also found 15 cis-eQTLs within mtDNA itself. Using these findings, they built genetic prediction models of mtDNA transcript levels, and used these predictions to test for assocition with quantitative traits and diseases in UK Biobank participants of European ancestry, indicating tissue specificity in mtDNA gene expression effects on complex traits and diseases. This is a comprehensive and well-executed study that nicely links molecular-level associations with complex phenotypes. I have the following comments:

1. mtDNA cis-eQTLs: most of the associations found in the mtDNA cis-eQTLs analyses have been found previously in Cai & Gomez-Duran et al 2020 Nature Medicine, please cite.

**Reply: We have now referenced this article in the section discussing mtDNA cis-eQTLs on page 6 as follows:**

**"In total, we found 15 cis-genome eQTLs (peak mtDNA genetic variant-gene expression pairs) for mtDNA-encoded transcript abundance after Bonferroni correction ($P<5x10^{-8}/15$, supplementary table 3), some of which have been previously observed[25]"**

2. Prediction of mtDNA encoded gene expressions: It would be good to see a figure of prediction r2 of a gene vs its r2 across tissues i.e. whether the accuracy of gene expression prediction is related to how correlated its predicted expression is across tissues. This matters for downstream TWAS studies where predicted mtDNA gene expressions are assessed for association with phenotypes, when for traits like bilirubin, there are ~40 associations with predicted mtDNA genes, presumably the same mtDNA genes in many different tissues due to the high levels of correlations between the predicted values.

**Reply: To test whether replication in phenotype association signals across tissues might arise from highly transferable prediction models, we examined the relationship between within-tissue predictive performance and cross-tissue generalisability. For each gene-tissue pair, we used the cross-validated $R^2$ generated from models (built with 80% of the data and tested in the remaining 20%) to quantify within-tissue performance and compared this to the mean predictive $R^2$ obtained when applying the corresponding full model to all other tissues. Contrary to the expectation that highly predictive models would generalise broadly, we observed a modest negative relationship, indicating that models performing best within their tissue of origin tended to transfer less well across tissues. This suggests that strong predictive performance could reflect tissue-specific regulation of mtDNA-encoded transcript abundance, rather than shared cross-tissue genetic effects, and therefore cross-tissue phenotype association signals are unlikely to be driven solely by redundant**

**prediction models. We have added this analysis and a corresponding figure (now Figure 2C) to the revised manuscript as per the below.**

**For the results section, we have included the following text to page 7:**

**"We also examined the relationship between within-tissue predictive performance and cross-tissue generalisability. For each gene-tissue pair, we compared cross-validated performance to the mean predictive performance obtained when applying the full model to all other tissues. We observed a negative relationship ($R=-0.26$, $P=8.4\times10^{-8}$), indicating that models with stronger predictive performance in their tissue of origin tended to generalise less well across tissues, perhaps suggesting tissue-specific genetic regulation of mtDNA transcript abundance rather than system-wide shared effects (Figure 2C)."**

**For the methods section, we have added the following text to page 22:**

**"To test the extent to which mtDNA transcript abundance prediction models generalise across tissues, we compared within-tissue predictive performance (described above) with cross-tissue transferability. For each mtDNA gene and tissue, the full prediction models were applied to estimate mtDNA transcript abundance in all other tissues for which data were available. Predictive accuracy in each external tissue was quantified as the coefficient of determination ($R^2$) between predicted and observed expression values after regressing out covariates as described above. For each gene-tissue pair, $R^2$ values were calculated for all nine models in each external tissue, the best performing model was selected, and cross-tissue transferability was summarised as the mean $R^2$ across all other tissues. Because cross-tissue performance was evaluated using models trained on the full dataset, these estimates are not fully out-of-sample and may be mildly optimistic. However, they provide a consistent measure of relative transferability across genes and tissues."**

3. TWAS of mitochondrial encoded genes: How should we understand the TWAS signals - the authors tested whether these signals are likely pleiotropic effects of the nuclear SNPs on a cis-regulated nuclear gene or the mtDNA gene, but not whether the nuclear SNPs are associated with the complex traits themselves (through some other mechanism than the cis-regulated nuclear gene). Since most GWAS hits are not cis-eQTLs, it is possible that the nuclear SNPs regulate the complex traits in a way that is not through the mtDNA genes, but not its cis-eQTLs either. I suggest the authors, at the very least, list the association of the nuclear SNPs with the complex traits or disease in question and discuss the possibility there are other indirect mechanisms in play.

**Reply: To evaluate whether the TWAS signals could reflect direct effects of nuclear genetic variants on the corresponding traits, we performed additional SNP-trait association analyses within our dataset as suggested. For each mtDNA prediction model significantly associated with a trait, we extracted up to the ten variants with the largest absolute SNP weights and tested their direct association with the same phenotype using identical individuals and covariate adjustments as in the TWAS analyses where possible. Across binary and quantitative traits, ~31% models contained at least one top-weighted SNP that remained significantly associated with the disease after multiple testing correction ($P<0.05/7448$ – the total number of genetic variants tested), indicating that only a subset of TWAS signals overlap with direct SNP-trait associations. These results suggest that some associations could involve alternative or pleiotropic pathways independent of mtDNA transcript abundance. However, direct SNP-trait association alone cannot distinguish mediation through mtDNA gene expression from parallel genetic effects and therefore does not exclude mtDNA-mediated mechanisms. We have now incorporated these results into**

**the manuscript and expanded the Discussion (page 17) to explicitly acknowledge the possibility of indirect or pleiotropic effects:**

**"Third, while the use of germline genetics provides evidence consistent with a directional link between predicted mtDNA transcript abundance and complex traits, we cannot fully exclude pleiotropic effects whereby genetic variants influence additional biological pathways. When testing direct trait associations for the top weighted variants contributing to each mtDNA prediction model significantly associated with a complex trait, at least one such variant was significantly associated with the corresponding trait in ~31% of cases across binary and quantitative trait models (P<0.05/7448, Supplementary tables 12 and 13). However, this analysis cannot distinguish mediation through mtDNA gene expression from parallel genetic effects, and therefore does not negate a potential role for mitochondrial regulation but highlights the need for cautious interpretation when inferring causality."**

**With the following added to the methods section on page 25:**

**"To evaluate whether significant associations between predicted mtDNA transcript abundance and complex traits could reflect direct effects of nuclear genetic variants on the corresponding traits, we performed additional genetic variant-trait association analyses. From each prediction model significantly associated with a complex trait, we extracted up to the ten genetic variants with the largest absolute weights (or all variants where fewer than ten were present), representing those contributing most strongly to predicted mtDNA transcript abundance. Each genetic variant, where available, was then tested directly for association with the corresponding phenotype using the same individuals and covariate adjustments as in the original analyses. Linear regression was applied for quantitative traits and logistic regression for binary traits using PLINK."**

4. TWAS effect sizes: some of the TWAS effect sizes are extraordinarily small e.g. 0.0000000001309716 for association between ENSG00000198840 in Artery Aorta (blup model) for C reactive protein, which gave a P value of 4.89E-11 in 300K samples (numbers taken from Supp Table S7). This doesn't look right to me, the authors need to check this and convince me this is right. In any case, the authors need to put in standard errors for the effect sizes. Other TWAS signal have slightly more credible yet still really small effect sizes, e.g. ENSG00000198899 in AdrenalGland has a effect of -0.0026332210 on C_reactive_protein_CRP with a P value of 2.54E-17. One reason is of course, these are not standardised effect sizes, but raw effect sizes (i.e. gene expression and complex traits both not scaled to mean 0, sd 1). What this would mean is tiny effects on gene expression of mtDNA genes are significantly associated with complex traits. If this was true, the authors would need to write about this explicitly and provide a plausible biological explanation how this can be.

**Reply: We thank the reviewer for raising this important point. The effect sizes originally reported in Supplementary Table S7 corresponded to raw, unstandardised regression coefficients, in which neither the genetically predicted mtDNA-encoded transcript abundance or the phenotypes were scaled to unit variance. As a result, the absolute magnitude of these coefficients is not directly interpretable and can be extremely small when the predicted transcript abundance has a narrow range, as is often the case for mtDNA-encoded genes whose prediction weights are individually small. In this setting, statistical significance is driven by the strength and consistency of the association across samples rather than the absolute scale of the regression coefficient.**

**To address this concern and improve interpretability, we have now re-run these analyses after standardising both genetically predicted mtDNA-encoded transcript abundance and quantitative phenotypes to mean zero and unit variance. We now report standardised effect sizes together with their corresponding standard errors in Supplementary Tables 8 and 10. These standardised estimates are directly comparable across genes, tissues and traits, and confirm that the reported associations are characterised by small but highly consistent effects.**

**During this reanalysis, we identified and corrected a labelling error affecting two phenotypes (waist-to-hip ratio and non-HDL), which led to a small number of previously reported associations being incorrectly attributed. After correction, the total number of Bonferroni-significant quantitative trait associations is 1,839 (previously 1,845). All affected tables, figures and text have been updated accordingly, and the overall patterns of association and the main conclusions of the study remain unchanged.**

5. TWAS tissues of interest: For traits like bilirubin where there are many tissues with the same associations, some are unlikely to be mechanistically related to bilirubin like the brain tissues. In other words, Fig 4 represents the statistically significant findings, but is likely an inflated view of how much mtDNA genes affect complex traits. I would ask the authors to include another figure (4b?) that plots the number of mtDNA genes associated rather than the total number of associations, to account for the number of redundant associations due to between tissues mtDNA gene expression correlations.

**Reply: To address this, we have now generated an additional summary showing the number of distinct mtDNA-encoded genes associated with each trait, rather than the total number of tissue-specific associations (Figure 4). The gene-level results broadly mirror the overall patterns observed in the tissue-based analysis, indicating that the reported trait associations are not driven solely by redundancy across correlated tissues. However, we note that mtDNA-encoded transcripts themselves are highly co-regulated and therefore collapsing across tissues introduces a different form of correlation among genes. As such, Figure 4 should be interpreted as a complementary summary that reduces tissue-level redundancy but does not represent fully independent signals across mtDNA genes.**

**We now describe this analysis in the Results section on (page 11):**

**"We also summarised the number of distinct mtDNA genes associated with each trait (Figure 4), and the resulting pattern closely mirrored the tissue-level analysis."**

**We have also edited the discussion section on page 18 to make this point clearer:**

**"Finally, in some cases, the tissues showing the strongest association with a trait may not be the most biologically intuitive. This could reflect shared regulatory variants across tissues (so that associations in one tissue may partly reflect effects in another, more relevant tissue), differences in sample size and statistical power, inter-individual expression variability across tissues or unknown aspects of tissue biology."**

6. Most of the mechanistic explanations given by the authors, for each group of traits associated with predicted mtDNA gene expression levels, goes along the lines of "this supports mtDNA involvement/influence on this trait/disease". This is fair enough given how little is known and the progress that is actively made by this paper to advance this. However, I would like to see a

discussion section on actual mechanistic speculations how mtDNA genes may mediate complex traits and diseases, and an outlook for future investigations.

**Reply: We thank the reviewer for this suggestion and have now outlined potential biological mechanisms in the manuscript. In brief, changes in mtDNA transcript levels could influence how efficiently mitochondria produce energy, because mtDNA encodes key components of respiratory chain complexes I, III, IV and V, whereas complex II is entirely nuclear encoded. This may affect cellular energy production, redox balance and mitochondrial signalling, with downstream consequences for metabolism and disease risk. Variation in mtDNA expression may also alter mitochondrial reserve capacity, particularly in tissues with high energy demand, potentially influencing responses to physiological stress. Finally, differences in transcript levels could modify the ability of cells to tolerate the accumulation of mtDNA mutations with age. These points have now been incorporated into the Discussion on page 16, together with an outlook describing future experimental approaches to test these hypotheses:**

**"Although our study does not directly resolve molecular mechanisms, several processes could link variation in mtDNA transcript abundance to complex traits and disease. mtDNA encodes essential subunits of respiratory chain complexes I, III, IV and V, but not complex II. Therefore, changes in mtDNA gene expression may alter oxidative phosphorylation efficiency, redox balance and mitochondrial signalling. Variation in mtDNA expression could also influence mitochondrial reserve capacity, particularly in energetically demanding tissues, potentially modifying cellular responses to physiological stress. In addition, differences in transcript abundance may affect the buffering capacity against the accumulation of mtDNA mutations with age. These processes may ultimately impair cellular function in a tissue-dependent manner, contributing to selective vulnerability of particular cell types. Pathological effects could arise directly through cell loss or indirectly through inflammatory responses triggered by the release of mitochondrial damage-associated molecular patterns. Future studies combining genetic perturbation of candidate nuclear regulators, functional characterisation of associated variants, and high-resolution transcriptomic and metabolic profiling will be important to clarify causal mechanisms linking mitochondrial gene expression to human phenotypes."**

7. Code is not complete on the github page, the scripts in the README do not match the name of the executable files, some executable files not present in github e.g. /scratch/grp/hodgkinsonlab/shared/GTEx_Data/Models/main.nf

**Reply: We thank the reviewer for identifying this issue. In the "methods/03.Genetic.Scores" section of the MitoX GitHub repository, the README file previously referenced a local version of the mitoimpute pipeline, including file paths that are not accessible outside our computing environment. This has now been corrected.**

***Reviewer #3 (Remarks to the Author):***

Summary: The authors leverage tissue-specific RNA and genome sequencing datasets to identify genetic variants associated with mitochondrially-encoded gene expression and disease risk. They identify 25 variants associated with mtRNA expression, 21 of which link to 5 nuclear genes involved in mitochondrial processes. Notably, 8 of the 25 associations involve FASTKD4, a nuclear-encoded protein that regulates mitochondrial RNA stability. The authors build machine learning models to predict mtRNA expression across tissues, construct genetic score models for

transcript abundance, and evaluate associations between predicted mtRNA expression and disease traits. They identify numerous trait-disease associations and explore quantitative trait associations using their genetic scores.

Overall, this work provides valuable new insights into the genetic regulation of mtRNA expression and its relationship to disease. However, several sections lack clarity, figures require further development, key results are missing from tables, and the overall take-home message needs to be articulated more clearly. More important methodological methods are not clearly explained. In parts, the lack of methods details has made it difficult to assess the quality of their analysis.

Major issues:

1. Incomplete presentation of variant effects across mitochondrial gene expression and in the affected nuclear genes.

For nuclear-encoded genes: The authors should demonstrate the effect of these variants on the expression levels of the associated nuclear genes. For example: Do the missense variants in FASTKD4 affect its total RNA levels? What about different FASTKD4 isoforms for the intronic variants? How do these changes explain the observed alterations in mitochondrial gene expression?

**Reply: Across the 25 significant nuclear-mtDNA associations identified, nine are driven by peak genetic variants that are missense mutations in nuclear genes with established mitochondrial roles (e.g. *FASTKD4*, *FASTKD5*). If these missense variants are indeed causal, we would not necessarily expect them to influence total RNA abundance or splicing of the nuclear gene itself. Instead, their effects are more plausibly mediated through altered protein function.**

**For the remaining 16 associations, we have now systematically examined GTEx v10 data to assess whether these variants act as *cis*-eQTLs or splice-QTLs for nearby nuclear genes in the relevant tissue. In 12 of these cases, the peak genetic variant was not significantly associated with expression or splicing of any nearby nuclear gene. In the remaining four cases, we observed significant *cis* associations with nearby nuclear genes, some of which are known to play a role in mitochondrial RNA processing (*FASTKD4*) and some that are not currently linked to these processes (e.g. *CCM2*, *MYOG*). It is important to note however, that as with all QTL-based analyses, the lead variant identified may not itself be causal, and incomplete power (particularly for splice-QTL detection) may obscure true regulatory effects on nearby nuclear genes.**

**We now report these new results on page 5 as follows:**

**"Finally, for peak genetic variants that were not protein-altering (16 associations), we assessed whether they act as *cis* expression or splice QTLs for nearby nuclear genes in GTEx v10. In four cases, we observed significant associations, including a genetic variant that is both an eQTL and sQTL for *FASTKD4* in tibial artery, consistent with its established role in mitochondrial RNA processing. The remaining associations involved nuclear genes without known mitochondrial functions (supplementary table 2), making them potential candidates for future functional studies to identify previously unrecognised nuclear regulators of mitochondrial processes."**

For mitochondrially-encoded genes: The manuscript does not clearly show which other mtDNA-encoded genes are affected by each identified variant. The authors state that "Considering all 350 possible variant–gene combinations (25 variants x 14 other mtDNA genes), we found that 58% showed nominal associations (P<0.05), suggesting a mixture of both broad and gene-specific regulatory effects." However, these results are not presented in either figures or supplementary tables. The authors have to show these results in the main figure and supplementary tables.

**Reply: We thank the reviewer for highlighting this and apologise for the omission of the underlying results. We have now included the full set of underlying results supporting this analysis. Specifically, we provide a comprehensive matrix of associations between each of the 25 peak nuclear variants and all mtDNA-encoded genes within the same tissue (25 variants x 14 additional mtDNA genes) in supplementary table 3, corresponding to the cross-gene comparison described in the text. In addition, associations between each peak genetic variant and the same mtDNA-encoded gene across all GTEx tissues are now reported in supplementary table 4, capturing the cross-tissue comparison. We now refer to these tables in the main text (page 5) at the point where these statistics are discussed.**

Moreover, the authors should relate their results to the literature on how effects in different nuclear encoded genes affect mtDNA expression. As an example, the authors could discuss how previous work support a model in which MTPAP and FASTKD4 can differentially affect different mtRNAs.

**Reply: We have now added text to the Discussion on page 15 (see below) linking our association results to prior mechanistic studies of nuclear-encoded regulators of mitochondrial RNA metabolism. In brief, our findings broadly mirror known functional specificity: loci encoding factors with wider roles in mitochondrial RNA metabolism (e.g. *FASTKD4*, *PNPT1* and *MRPS35*) were associated with multiple mtDNA transcripts, whereas *FASTKD5*, which has a more transcript-specific role, showed a focused association primarily with *MTCO1*:**

**"For instance, variants near *MRPP3*, a catalytic subunit of the mitochondrial RNase P complex, were associated with transcript levels of multiple mtDNA genes, consistent with its role in cleaving precursor transcripts. Similarly, variants associated with factors with wider roles in mitochondrial RNA metabolism, including *FASTKD4*, *PNPT1* and *MRPS35*, were linked to multiple mtDNA transcripts, consistent with their broad effects on mitochondrial gene expression, whereas *FASTKD5* showed a more restricted association predominantly with *MTCO1*, in line with its more transcript-specific role[23,28,40]."**

Regarding tissue-specific differences: It is not clear which biological conditions might give rise to the observed diversity in response to genetic variants. Are the differences across tissues related to differential effects on the expression levels of the associated nuclear genes? For example, do different tissues show different effects on mtRNA gene expression levels?

**Reply: Tissue-specific differences in the association between genetic variation and mtDNA-encoded transcript abundance may arise from several factors. First, regulatory variants may act through tissue-specific elements, such as enhancers active only in particular cellular contexts. Second, the functional importance of nuclear-encoded mitochondrial regulators may differ across tissues with distinct metabolic and physiological demands, potentially leading to genuine tissue-specific effects on mtDNA transcript abundance. Finally, differences in sample size, expression variability and statistical power across GTEx tissues**

**are also likely to contribute to heterogeneity in observed effect sizes and significance. In line with this, we have added the following text to the Discussion (page 15):**

**"We also observe some tissue-specific associations, which may reflect regulatory variants acting through tissue-restricted mechanisms, including differences in cell-type-specific transcriptional environments, or differences in the functional relevance of nuclear-encoded mitochondrial regulators across tissues with distinct metabolic and physiological demands."**

In Figure 1B the authors should provide a visual representation of statistical significance (e.g., p-values indicated by symbols or color intensity). Moreover, the authors do not explain why some tissues show increased MTATP6 levels (e.g., Colon) while others show reduced levels (e.g., Muscle) for the same variant. This directional variability across tissues requires explanation.

**Reply: We have now updated Figure 1B to include colour coding that distinguishes associations significant after Bonferroni correction, nominally significant associations (P<0.05) and non-significant results.**

**Regarding the apparent directional variability across tissues for *MTATP6*, most tissue-specific estimates are not statistically significant and therefore reflect sampling variation around zero rather than true opposite biological effects. Only the association in subcutaneous adipose tissue remains significant after multiple-testing correction and shows reduced *MTATP6* expression. A small number of additional tissues show nominal associations, the majority in the same direction of effect, while the single opposite-direction nominal signal does not withstand correction for multiple testing and is therefore likely to represent statistical noise.**

**To clarify this point, we have revised the relevant text in the Results on page 5 to include:**

**"For example, the association between rs12579998 and MTATP6 was largely restricted to adipose tissue, with additional nominal associations (P<0.05) observed in only four other tissues, one of which showed an opposite direction of effect, although this did not withstand multiple-testing correction and is therefore likely to reflect statistical variation given the available sample sizes (Figure 1B)."**

Finally, as different tissues have different levels of expression of individual mtRNAs (Mercer et al 2011), the authors should discuss how this tissue variability can affect their results. Moreover, they should consider this in their methods when comparing gene expression differences across tissues (see below).

**Reply: While baseline expression levels of individual mtDNA-encoded transcripts differ across tissues, mtDNA genes are highly and robustly expressed in all GTEx tissues analysed, reflecting the abundance of mitochondria across cell types. Importantly, eQTL detection is performed within each tissue independently and depends on inter-individual variation in expression rather than absolute expression levels. As a result, cross-tissue differences in baseline mtDNA-encoded transcript abundance do not bias association testing or generate spurious eQTLs.**

**We now clarify this point in the Methods section (page 20) as follows:**

**“Although baseline expression of mtDNA-encoded transcripts varies across tissues, these genes are highly expressed across all tissues analysed, and association detection depends on inter-individual variability within tissues rather than absolute expression levels.”**

2. Machine Learning Models: Insufficient methodological detail and integration with the biology discussed in the paper.

The machine learning section lacks critical details about model construction and interpretation. The authors state they used LASSO, Elastic Net, and BLUP to build predictive models, selecting genetic variants "based on different P-value thresholds." However, the Methods section does not clarify which features were used as model inputs, whether or how features were scaled before training, how model optimization was performed and the final feature coefficients in each model. Moreover, since LASSO and Elastic Net are interpretable algorithms where feature importance relates directly to coefficients, it is essential to report which features are important and their relative weights.

**Reply: The predictive models were built using all genetic variants passing the specified P-value thresholds (all genetic variants, $P<0.01$, $P<1\times10^{-5}$) as features, as described in the Methods. Feature scaling, regularisation and model fitting are handled internally by FUSION[48] (variant dosages are standardized, LASSO and Elastic Net apply regularization to select informative features while controlling overfitting and BLUP models estimate polygenic effects as random effects). Due to the very large number of models (49 tissues x 15 genes x 3 P-value thresholds x 3 machine learning methods), reporting individual feature coefficients for all models in the manuscript is not feasible. However, all SNP weights for each gene, tissue, method and P-value threshold are made available in the data release (see Data Availability), allowing any user to inspect feature importance for individual models. We note that the eQTL analyses in the manuscript already highlight the variants with the strongest biological effects, which are the primary drivers of the predictive models. We have now added the following to the methods section on page 21 to make this clearer:**

**“In FUSION, variant dosages are standardised prior to model fitting, LASSO and Elastic Net apply regularisation to select informative features while controlling overfitting and BLUP estimates polygenic effects as random effects”**

In Figure 2C, the correlation between internal and external $R^2$ values appears to show two distinct populations of points. This pattern is not discussed.

**Reply: While the scatter shows variability in model performance, we do not observe clear evidence for two discrete populations. Rather, the apparent separation likely reflects heterogeneity in predictive performance across genes and tissues. Differences in the genetic predictability of mtDNA-encoded transcript abundance, along with variation in sample size and expression variability across tissues, can lead to some gene-tissue models performing consistently well in both internal and external validation, whereas others show weaker predictive accuracy and therefore appear separated in the plot. We have added the following sentence to the Results (page 8) to clarify this point:**

**“$R^2$ values are significantly correlated between internal and external validation tests ($P=0.009$, Pearson’s $R=0.38$, Figure 2D), although predictive performance varies across genes and tissues, with some models showing consistently stronger performance and others weaker generalisation.”**

The authors should expand on their interpretation of the results. The could explain how the results from the machine learning models relate to the downstream disease association analyses or the biology of the mtDNA expression.

**Reply: We thank the reviewer for this suggestion. In response, we have expanded the Discussion on page 16 to clarify how genetically predicted mtDNA transcript abundance relates to downstream disease associations and the underlying biology. As described in our response to Reviewer 1 (point 6), we now outline potential biological mechanisms linking variation in mtDNA transcript abundance to complex traits and disease. For completeness, the added text is reproduced here:**

**"Although our study does not directly resolve molecular mechanisms, several processes could link variation in mtDNA transcript abundance to complex traits and disease. mtDNA encodes essential subunits of respiratory chain complexes I, III, IV and V, but not complex II. Therefore, changes in mtDNA gene expression may alter oxidative phosphorylation efficiency, redox balance and mitochondrial signalling. Variation in mtDNA expression could also influence mitochondrial reserve capacity, particularly in energetically demanding tissues, potentially modifying cellular responses to physiological stress. In addition, differences in transcript abundance may affect the buffering capacity against the accumulation of mtDNA mutations with age. These processes may ultimately impair cellular function in a tissue-dependent manner, contributing to selective vulnerability of particular cell types. Pathological effects could arise directly through cell loss or indirectly through inflammatory responses triggered by the release of mitochondrial damage-associated molecular patterns. Future studies combining genetic perturbation of candidate nuclear regulators, functional characterisation of associated variants, and high-resolution transcriptomic and metabolic profiling will be important to clarify causal mechanisms linking mitochondrial gene expression to human phenotypes."**

3. Age-related disease associations: The section "Associations with age-related common diseases" discusses important findings but lacks accompanying figures or comprehensive tables. The authors should provide a figure or table summarizing these associations for easier interpretation.

**Reply: We thank the reviewer for pointing this out and apologise for the lack of a clear summary. We have now addressed this in two ways. First, we have added Table 3 to the main manuscript, which summarises all transcriptome-wide significant mtDNA gene-tissue associations for the five age-related diseases examined. Second, we have added a new supplementary table 11 that provides the full set of TWAS results for all mtDNA genes, tissues, prediction models and diseases tested. Together, these additions provide a concise overview of the significant findings while allowing comprehensive inspection of all results.**

4. RNA sequencing analysis
The RNA sequencing analysis lacks critical detail about how the libraries were constructed. In page 19 the authors say: "Second, although we model gene expression separately for each of the 15 mtDNA-encoded genes, mitochondrial transcription operates via polycistronic mechanisms. Some genes (e.g., MTATP6/8) are bicistronic, and MTND6, transcribed from the light strand, is not polyadenylated and thus should be underrepresented in GTEx poly-A+ data. Similarly, mitochondrial rRNAs are minimally adenylated and typically excluded from standard RNA-seq. Nevertheless, we find robust signals for several of these genes, which may reflect alternative processing (e.g., degradation fragments or longer precursor transcripts) and still carry informative biological signal."

Are the GTEx libraries polyA-selected libraries? In that case, the authors should emphasize this point more strongly and clearly and tone down their conclusions. It is not clear whether the levels of these transcripts represents a difference in the library quality rather than a real biological difference.

How are the authors controlling for library depth differences across tissues? Do they observe any bias? Are they excluding outlier samples? PCA analysis and other quality metrics should be presented in a supplementary figure.

**Reply: GTEx RNA-seq libraries are generated using poly(A) selection, and we agree that this can influence the representation of certain mitochondrial transcripts, particularly non-polyadenylated or weakly adenylated RNAs such as *MTND6* and mitochondrial rRNAs. We have clarified this point in the manuscript and tempered the wording accordingly (see below). Importantly, because library preparation and sequencing protocols are consistent across samples within each GTEx tissue, poly(A) selection affects overall transcript abundance but does not create genotype-dependent differences and therefore does not generate spurious eQTLs. Instead, reduced capture efficiency for some transcripts is expected primarily to reduce statistical power, perhaps leading to false negatives, but not false positives.**

**We control for technical variation in several ways. Gene expression values were normalised to transcripts per million (TPM) to account for library size, and extensive RNA-seq quality control was applied, including removal of samples with low read depth, abnormal mapping rates or other QC failures. We additionally removed visual outliers based on gene expression distributions and principal component analysis, as described in the Methods. We did not observe systematic biases suggestive of library-quality driven effects.**

**To clarify these points, we have expanded the Methods and Discussion (pages 18 and 17, respectively) to explicitly note the use of poly(A) libraries, the expected underrepresentation of some mitochondrial transcripts, and the fact that this is more likely to affect sensitivity than to introduce false biological signal, as follows:**

**"GTEx RNA-seq libraries are generated using poly(A) selection, which enriches for polyadenylated transcripts and may reduce capture of some mitochondrial RNAs, particularly weakly or non-polyadenylated species."**

**"Nevertheless, reduced capture of weakly or non-polyadenylated mitochondrial transcripts is expected to affect sensitivity rather than introduce genotype-dependent bias in association analyses, and we find robust signals for several of these genes, which may reflect alternative processing (e.g., degradation fragments or longer precursor transcripts) and retain informative biological signal."**

For the RNA sequencing analysis, the authors take alignment files, convert them back to fastq files, trim, and realign them. What is the reason for this? The authors do not have access to raw fastq files? This procedure can be problematic as part of the original data might be lost in the first alignment step, to which the authors' approach is unaware of. Did the authors make sure their fastq files are similar to the raw data from GTEx? The authors should discuss this in their methods section.

**Reply: Raw FASTQ files are not available for download from the GTEx dataset. GTEx provides BAM files that contain all original reads, including unmapped reads. To ensure consistency in data processing across all samples, we converted these BAM files back to FASTQ, trimmed and realigned them. This approach is therefore effectively equivalent to starting from raw sequencing data and preserves the original read content. We have clarified this rationale in the Methods section on page 18 to address potential concerns about data loss during the initial alignment, as follows:**

**"As raw FASTQ files are not available from GTEx, BAM files containing all original reads (including unmapped reads) were converted back to FASTQ using samtools[37] (v1.14). This approach allows consistent trimming and realignment across all samples and is effectively equivalent to starting from raw sequencing data."**

Given that different tissues have different numbers of samples and RNA sequencing depth, the authors should perform a power analysis to understand how much of the difference they see across tissues might arise from this fact rather than a biological difference.

**Reply: We agree that differences in sample size and RNA-sequencing depth across GTEx tissues influence statistical power to detect eQTLs and may contribute to variation in the number of significant associations observed across tissues. This is an expected feature of multi-tissue studies and affects sensitivity rather than the validity of detected associations. In our analysis, each tissue was tested independently using its available sample size, and we report sample numbers and genomic inflation factors for all tissues (Supplementary Table 1) to allow readers to assess differences in statistical power.**

**Importantly, mtDNA-encoded transcripts are highly expressed across all tissues, reducing the likelihood that differences in detection are driven by expression dropout, and suggesting that variation in sample size and inter-individual expression variability are the primary contributors to tissue-specific sensitivity. While a formal power analysis is challenging given the complex multi-tissue design and variable genetic architectures, we now explicitly acknowledge in the Discussion (page 17) that differences in power across tissues may influence detection sensitivity and interpretation of tissue specificity:**

**"Finally, in some cases, the tissues showing the strongest association with a trait may not be the most biologically intuitive. This could reflect shared regulatory variants across tissues (so that associations in one tissue may partly reflect effects in another, more relevant tissue), differences in sample size and statistical power, inter-individual expression variability across tissues or unknown aspects of tissue biology."**

Minor issues:
The statement "These associations point to RNA processing, rather than transcription initiation alone, as a major determinant of mtRNA abundance" appears to overlap with findings from previous work. Please clarify the novel contribution here.

**Reply: We thank the reviewer for this comment. We agree that prior experimental studies have established an important role for mitochondrial RNA processing in shaping mtRNA abundance. Our intention was not to suggest that this mechanism is newly discovered, but rather to highlight that our population-scale human genetic data provide independent *in vivo* evidence supporting RNA processing as a major determinant of mtRNA levels across tissues. Unlike previous mechanistic studies, our analysis identifies naturally occurring nuclear genetic variation affecting mitochondrial RNA-processing genes and demonstrates**

**their impact on mtDNA transcript abundance across multiple human tissues. We have clarified this point in the manuscript (page 15) to better distinguish prior biological knowledge from the novel contribution of our study:**

**"Consistent with prior experimental studies, these associations provide population-scale human genetic evidence that RNA processing, rather than transcription initiation alone, is a major determinant of mtRNA abundance across tissues."**

The authors highlight a number of genes known to function within the mitochondria, including FASTKD4, FASTKD5, PNPT1, and MTPAP which they claim are linked to associations listed in Table 1. The text lacks citation to any literature on these genes to support the author's description of gene function. Citation would be especially useful for the statement "PNPT1 is responsible for the import of RNA into the mitochondria", as import of RNA into human mitochondria is highly contested. Additionally, the text later refers to LONP1 as being involved in mitochondrial RNA processing, but does not comment on the known role of LONP1 as a mitochondrial protease.

**Reply: We have now included citations for all gene functions in this section, and have clarified the roles of PNPT1 and *LONP1* as follows:**

**"*PNPT1* is implicated in mitochondrial RNA metabolism and, in some experimental systems, RNA import into mitochondria[24]"**

**"Applying the same functional annotation approaches as above, we linked peak nuclear genetic variants to several known mitochondrial RNA processing genes, including *MRPP3* and *ELAC2*[28], the mitochondrial protease *LONP1*[29], as well as other genes with unknown links to mitochondria that may warrant further investigation (*MYOG*, *NACAD*, *REP15* and *CCM2*)."**

***Reviewer #4 (Remarks on code availability):***

I reviewed the code used for RNA sequencing as this is my area of expertise. The script provided is a perl script that generates a bash script that runs the pipeline from bam to fast conversion to alignment and sorting. The scripts is well structured and clear for an individual that is familiar with these processes but it will benefit from more comments to help users less familiar with the general RNA sequencing analysis.

**Reply: We are grateful to the reviewer for their careful evaluation of the RNA-sequencing pipeline and for noting that the script is well structured. In response to the suggestion, we have added additional explanatory comments throughout the script to improve clarity and accessibility for users less familiar with RNA-sequencing data processing.**

**Additional minor correction:**

**During revision, we identified and corrected an error in the reported SNP identifier in the Results section describing the MTND4-breast cancer association. The variant previously listed as rs1078806 was incorrectly labelled and has been corrected to rs10736303. This change does not affect any analyses, results or conclusions. The correction is clearly marked in the revised manuscript.**

**Editorial Changes:**

Abstract must be 150 words or fewer

**Reply: We have now edited the abstract to be 150 words or fewer.**

We thank the reviewers for their further constructive comments on our manuscript. Below, we provide a detailed, point-by-point response outlining all changes made.

REVIEWER COMMENTS

***Reviewer #1:***

No summary stats, it says "to come".

**Reply: Filtered summary statistics (P < 0.05) have now been uploaded to Figshare to support use by the community, as described in the 'Data Availability' section of the manuscript.**

***Reviewer #2:***

Making SNP weights available in the data release is a welcome step, but Supplementary Table 13 lacks column descriptions. A brief legend should be added so future researchers can interpret these results.

**Reply: We have now expanded the table legend to include detailed descriptions of all columns and clarify the structure and interpretation of the results.**

The expanded Discussion addresses potential biological mechanisms in general terms but does not connect them to specific ML model results. The authors are encouraged to include one or two concrete examples linking a model outcome to a biological inference (e.g., which gene, in which tissue, motivates a given mechanistic interpretation).

**Reply: We thank the reviewer for this helpful suggestion. We agree that explicitly linking specific model results to biological interpretation improves clarity. While the genetic scores reflect the combined effects of multiple nuclear variants and are not readily attributable to single genes, we have now included illustrative examples in the Discussion to connect specific tissue-trait associations to potential mechanisms. In particular, we highlight the association between predicted MTND1 transcript abundance in aorta and hypertension, and briefly relate additional associations in neural tissues to neurodegenerative traits (page 17), as follows:**

**"Although our study does not directly resolve molecular mechanisms, several processes could link variation in mtDNA transcript abundance to complex traits and disease. mtDNA encodes essential subunits of respiratory chain complexes I, III, IV and V, but not complex II. Therefore, changes in mtDNA gene expression may alter oxidative phosphorylation efficiency, redox balance and mitochondrial signalling. For example, in our analysis, the association between predicted MTND1 transcript abundance in aorta and hypertension may reflect altered oxidative phosphorylation efficiency in vascular smooth muscle, potentially influencing vascular tone and remodelling. Similarly, associations observed in neural tissues for neurodegenerative traits may reflect the high energetic demands and mitochondrial vulnerability of these cell types."**

Finally, if the primary contribution of this paper is a model demonstrating that mtRNA levels can be predicted from genetic information, then the full model with tissue-specific weights must be made publicly available in a usable form, ideally via GitHub with sufficient documentation to allow retraining and reuse. The current GitHub page is incomplete and does not provide a clear path for reproducing the results. This should be addressed before publication.

**Reply: We have now expanded the GitHub repository to improve clarity and usability. Specifically, we have added detailed documentation describing the structure of the score files (including column definitions and allele alignment), provided guidance on applying the models to external genotype data and clarified the differences between available models and their relationship to the manuscript results. We have also included example commands and output files to facilitate reuse.**
